# A systematic review and meta-analysis to evaluate the diagnostic accuracy of recognition of stroke in the emergency department (ROSIER) scale

**DOI:** 10.1186/s12883-020-01841-x

**Published:** 2020-08-18

**Authors:** Fei Han, Chao Zuo, Guodong Zheng

**Affiliations:** 1 Department of Emergency, Beicheng Hospital of Chinese Medicine Hospital of Linyi City, Wuhushan road, Lanshan district, Linyi City, Shandong province China; 2 Shandong Medical College, Linyi City, Shandong Province China; 3 Department of Neurosurgery, Linyi City People Hospital, Linyi City, Shandong Province China

**Keywords:** Validation studies, Review, meta-analysis, ROSIER, stroke

## Abstract

**Background:**

The present study aims to evaluate the performance and the clinical applicability of the Recognition of Stroke in the Emergency Department (ROSIER) scale via systematic review and meta-analysis.

**Methods:**

Electronic databases of Pubmed and Embase were searched between 1st January 2005 (when ROSIER developed) and 8th May 2020. Studies that evaluated the diagnostic accuracy of the ROSIER scale were included. The sensitivity, specificity, diagnostic odds ratio (DOR), and area under the curve (AUC) were combined using a bivariate mixed-effects model. Fagan nomogram was used to evaluate the clinical applicability of the ROSIER scale.

**Results:**

A total of 14 studies incorporating 15 datasets were included in this meta-analysis. The combined sensitivity, specificity, DOR and AUC were 0.88 [95% confidence interval (CI): 0.83–0.91], 0.66 (95% CI: 0.52–0.77), 13.86 (95% CI, 7.67–25.07) and 0.88 (95% CI, 0.85–0.90), respectively. Given the pre-test probability of 60.0%, Fagan nomogram suggested the post-test probability was increased to 79% when the ROSIER was positive. In comparison, it was decreased to 22% when ROSIER was negative. Subgroup analysis showed that the pooled sensitivity of ROSIER in the European population was higher than that in Asia. In contrast, the pooled specificity was not significantly different between them. Moreover, results also suggested the male-to-female ratio ≤ 1.0 subgroup, prehospital setting subgroup, and other trained medical personnel subgroup had significantly higher sensitivity compared with their counterparts. At the same time, no significant differences were found in the pooled specificity between them.

**Conclusions:**

ROSIER is a valid scale with high clinical applicability, which has not only good diagnostic accuracy in Europe but also shows excellent performance in Asia. Moreover, the ROSIER scale exhibits good applicability in prehospital settings with other trained medical personnel.

## Background

Stroke is a severe concern in the emergency department and remains the leading cause of death and disability [1, 2]. Early identification of patients with stroke and providing thrombolysis therapy can reduce morbidity and mortality [3, 4]. However, due to the misdiagnosis and inappropriate triage, many patients missed the best time for treatment [5–7]. Thus, a series of screening tools had been developed to help emergency physicians to conduct a rapid and accurate diagnosis of stroke [8–11]. The Recognition of Stroke in the Emergency Department (ROSIER), which was developed by Nor and colleagues in 2005, is one of the commonly recommended stroke scales in the western world [7].

ROSIER is a 7-item recognition instrument (ranging from − 2 to + 5) that based on the clinical history and neurological signs. A score of + 1 or above was considered positive of stroke or transient ischemic attack [7]. During the past decades, several studies have been conducted to validate the diagnostic accuracy of ROSIER in different countries and work settings, but the results were not consistent [12–17]. Although previous studies have systematically evaluated its performance [18–21], the clinical utility and the applicability in other countries, and investigators have not been investigated before. Moreover, another seven studies have not been incorporated in previous meta-analyses [22–28].

In the present study, we aim to conduct a systematic review and meta-analysis to evaluate the diagnostic accuracy and clinical applicability of the ROSIER scale. Additionally, we also aim to discuss its performance in Asia, prehospital setting, and other trained medical personnel.

## Methods

### Literature search strategy

The terms of “stroke” OR “brain ischemic” OR “transient brain ischemia” OR “cerebra arterial disease” OR “non-ischemic stroke” OR “ischemic stroke” OR “cerebrovascular accident” OR “intracranial artery disease” AND “Recognition of Stroke in the Emergency Room” OR “ROSIER” were searched as medical subject headings (MeSH) in the Pubmed and Embase database for all the articles concerning the validation of the ROSIER model between 1st January 2005 (the ROSIER was developed) and 8th May 2020. The references were also manually checked for relevant papers.

### Inclusion and exclusion criteria

Publications included in the present meta-analysis fulfill the criteria of (1) written in English; (2) use image logical examination as the golden standard for stroke diagnosis; (3) provide sufficient information for calculating true positive (TP), false positive (FP), false negative (FN) and negative (TN); (4) with a threshold as> 0. When multiple publications concerned about the same population, the most complete or updated one was included.

### Data abstraction

Characteristics of the first author, publication year, geographic background, study design (prospective or retrospective), work setting (emergency department or prehospital settings), ROSIER assessment investigator (emergency physicians, or other medical personnel), study period, sample size, mean age or rang of age, TP, FP, FN, and TN were independently extracted by two investigators. Any discrepancies were resolved by consensus.

### Statistical analysis

The pooled sensitivity, specificity, and diagnostic odds ratio (DOR) were calculated using a bivariate mixed-effects model. DOR is the risk ratio in stroke relative to that in the control group [29]. The pooled sensitivity and specificity data were used to construct the summary receiver operating characteristic curve (SROC), and the area under the curve (AUC) was used for evaluating the performance of the ROSIER scale [30]. *I*^2^ measure the heterogeneity among the studies. The value of < 50% was considered as no heterogeneity. A sensitivity analysis was conducted to assess the effect of each dataset on the performance by sequentially omitting each data set [31]. The quality of methodology in each study was evaluated by the two investigators using the Quality Assessment of Diagnostic Accuracy Studies (QUADAS) [32]. Subgroup analysis was used for stratifying the studies by geographic background, study design, study setting, type of investigator, sample size, male-to-female ratio, and study quality. The trends of the diagnostic odds ratio (DOR) by ranking the publication year, sample size, and study quality were analyzed using cumulative meta-analyses. Publication bias was detected by Deek’s funnel plot, using 1/root (effective sample size) versus the log DOR. *P* < 0.05 for the slope coefficient indicates significant asymmetry [33]. Clinical applicability of the ROSIER scale was evaluated by the Fagan nomogram, which was constructed by using the positive likelihood ratio and negative likelihood ratio [34].

Pooled sensitivity, specificity, SROC, DOR, and Fagan nomogram were conducted using Stata statistical software version 14.0 (StataCorp, College Station, TX). Cumulative meta-analysis was conducted by Comprehensive Meta-Analysis version 2.0 (Biostat, Englewood, NJ, USA). All the statistical significance levels were set two-tailed at *p* < 0.05.

## Results

### Characteristics of the included studies

**Fig. 1:**
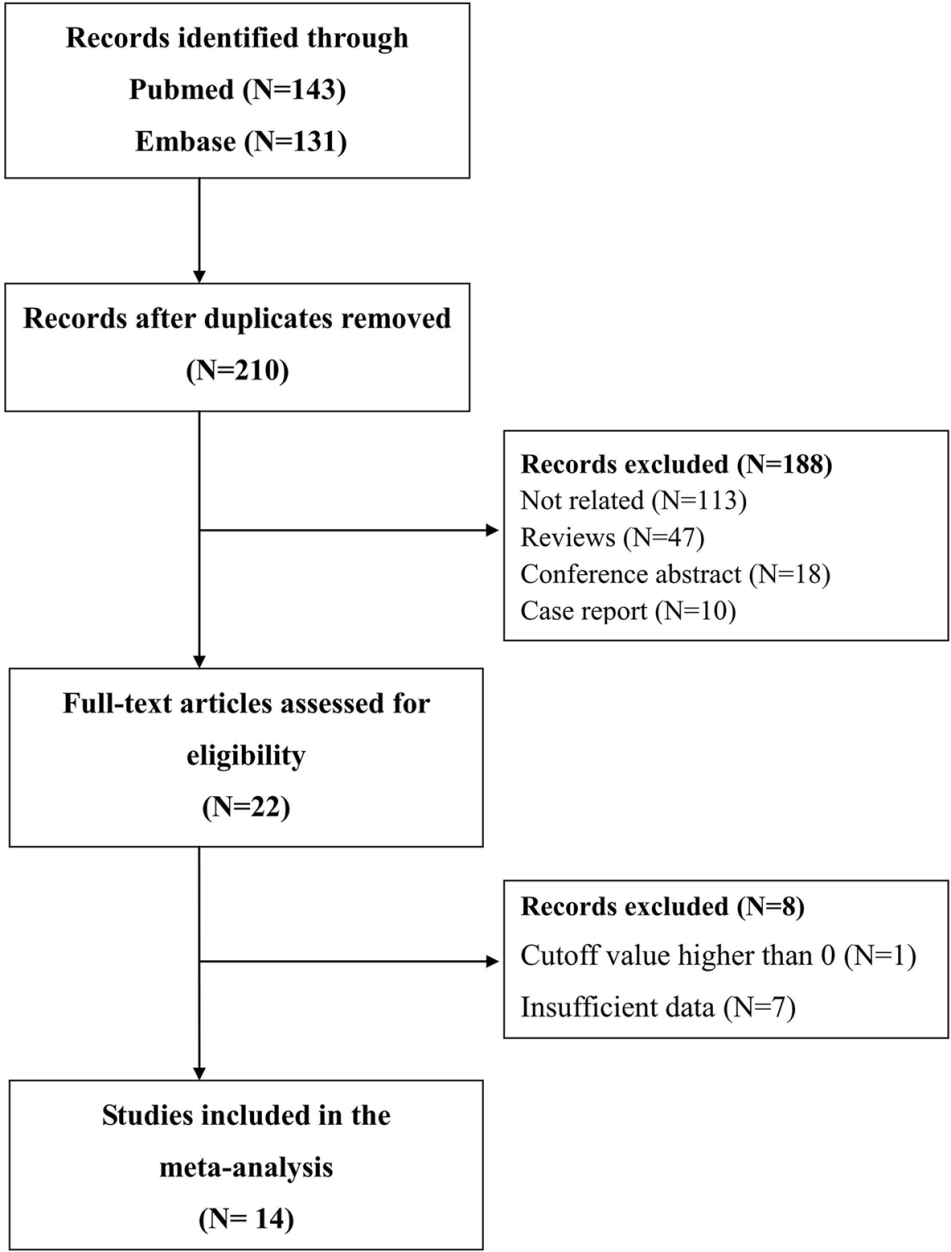
The flow-chart of the study selection for estimating the diagnostic accuracy of the Recognition of Stroke in the Emergency Department scale

A total of 274 articles were acquired from the electronic databases. After a full examination, 159 publications were finally excluded: 64 were duplicated, 113 were not related, 47 were reviews, 18 were conference abstract, 10 were case report, one did not use a cutoff value of four [35] and seven not provided sufficient data [36–42]. (Fig. 1) In the end, a total of 14 studies with 15 datasets were included in this meta-analysis. Among them, five were conducted in United Kindom [7, 13, 15, 22, 24], four in China [14, 16, 27, 28], one in Korea [17], one in Portugal [23], one in Germany [25], one in Ireland [12] and one in Australia [26]. The characteristics of the included studies are shown in Table 1.

**Table 1:** Characteristics of the studies included in this systematic review and meta-analysis

Ref	First Author	Publication Year	Country	Design	site	Investigator	Study population	Time period	Sample size	Mean age (years)	TP	FP	FN	TN
[7]	Nor-1	2005	United kingdom	Prospective	emergency room	emergency physicians	Subjects aged older than 18 years with suspected stroke or transient ischaemic attack (TIA) in the emergency room were consecutively assessed during a 1-year period in Newcastle Hospital	2001/8/1~ 2002/7/31	343	70.49 (stroke 70 ± 14; no-stroke 71 ± 16)	162	23	14	144
[7]	Nor-2	2005	United kingdom	Prospective	emergency room	emergency physicians	Subjects aged older than 18 years in 9-month prospective cohort study with suspected stroke or TIA with symptoms or signs in the emergency room in Newcastle Hospital	2002/11/1~ 2003/7/31	160	71.37 (stroke 71 ± 14; no-stroke 72 ± 16)	94	10	7	49
[12]	Jackson	2008	Ireland	Prospective	emergency department	emergency physicians	Consecutive patients identified on routine initial triage as having possible or suspected stroke admitted to the St James’s hospital emergency department	NA	50	73 (24–91)	44	3	2	1
[22]	Byrne	2011	United Kingdom	Prospective	stroke unit	registered trained nurses	A prospective audit of patients with a suspected stroke or transient ischemic attack (TIA) were admitted to the stroke unit of an acute hospital over an eight month period in Northern Ireland	2008/7~ 2009/2	100	69.89 (stroke 72 ± 14.6; no-stroke 64 ± 21)	69	14	3	14
[13]	Whiteley	2011	United Kingdom	Prospective	emergency department	neurologist	Consecutive patients with suspected acute stroke who presented to the emergency department of the Western General Hospital, Edinburgh while the study neurologist was available	2007/3/21~ 2009/2/27	356	72 ± 14 (stroke 74 ± 13; no-stroke 67 ± 16)	203	62	43	48
[23]	Gregório	2012	Portugal	Prospective	specialist neurovascular clinic	attending neurologist	Consecutive patients with suspected TIA or minor stroke referred over one year to a specialist neurovascular clinic in a university hospital.	NA	779	70	356	212	58	153
[14]	He	2012	China	Prospective	pre-hospital setting	emergency physicians	All patients aged older than 18 years old with suspected stroke or TIA with symptoms or signs seen by emergency physician in emergency department the Foshan Hospital of Traditional Chinese Medicine (FSTCM)	2010/4~ 2011/11	540	63 (18–96)	341	27	38	134
[24]	Benjamin	2013	United kingdom	Retrospective	NA	neurologist physician	People with HIV who had a stroke or a stroke mimic at the Royal Liverpool University Hospital and North Manchester General Hospital	2007/1~ 2009/12	56	38.80 stroke 40.5 (32–46) no-stroke 38 (33–46)	13	12	5	26
[15]	Fothergill	2013	United kingdom	Prospective	ambulance setting	ambulance clinicians	Patients presented with symptoms of stroke, were assessed by participating ambulance clinicians and conveyed to the Royal London Hospital.	2010/1/4~ 2011/3/31	295	65 (20–95)	171	97	6	21
[16]	Jiang	2014	China	Prospective	emergency department	Stroke nurses or a consultant in emergency medicine	Patients all patients with suspected stroke or transient ischaemic attack (TIA) in the emergency department of the Prince of Wales Hospital (PWH), a tertiary referral center affiliated with the Chinese University of Hong Kong	2011/6/1~ 2011/12/31	715	70.56 (stroke 72 ± 13; no-stroke 69 ± 14)	323	203	48	141
[17]	Lee	2014	Korea	Prospective	emergency department	emergency physicians	Patients with suspected acute stroke who were admitted to emergency department	2013/8~ 2014/2	312	60	100	15	11	186
[25]	Purrucker	2015	Germany	Retrospective	emergency room	emergency medical services paramedics and emergency physicians	Consecutive cases of preclinically ‘suspected central nervous system disorder’ admitted to the emergency room (ER) of the Heidelberg University Hospital	2007/11/1~ 2010/8/31	640	61.7 ± 20.9 (stroke 75.6 ± 13.4; no-stroke 56.0 ± 20.8)	144	96	36	364
[26]	MacKay	2016	Australia	retrospective and prospective	emergency department	emergency physicians	The patient population consisted of a prospective cohort of children aged 1 month to 18 years with mimics, and a mixed prospective/retrospective cohort of children aged 1 month to 18 years with arterial stroke (AIS) and hemorrhagic stroke (HS) at the Royal Children’s Hospital Melbourne, a tertiary Australian pediatric referral center	stroke: 2003 ~ 2010 non-stroke: 2009–2010	380	9.6 (4.6–13.7) stroke 7.8 (3.6–12.2) non-stroke 10.2 (5.2–13.9)	68	86	33	193
[27]	Mao	2016	China	prospective	emergency department	emergency doctor	Patients with suspected stroke in emergency department of the second Affiliated Hospital of Guangzhou Medical University (AHGZMU)	2012/5~ 2013/03	416	69.40 (stroke 69.2 ± 13.8; no-stroke 70.6 ± 11.4)	278	17	80	41
[28]	He	2017	China	prospective	emergency medical service	general practitioners	All patients with suspected stroke or TIA and with symptoms or signs observed by general practitioners in the Luocun Community Health Service Center (LCHSC) of Nanhai District and Zhangcha Community Health Service Center (ZCHSC) of Chancheng District, Foshan City	2012/8~ 2016/1	468	67.54 ± 12.66 (stroke 71.32 ± 11.50)	276	26	56	110

*NA* not available, *TP* true positives, *FN* false negatives, *FP* false positives, *TN* true negatives

### Diagnostic accuracy of the ROSIER

**Fig. 2:**
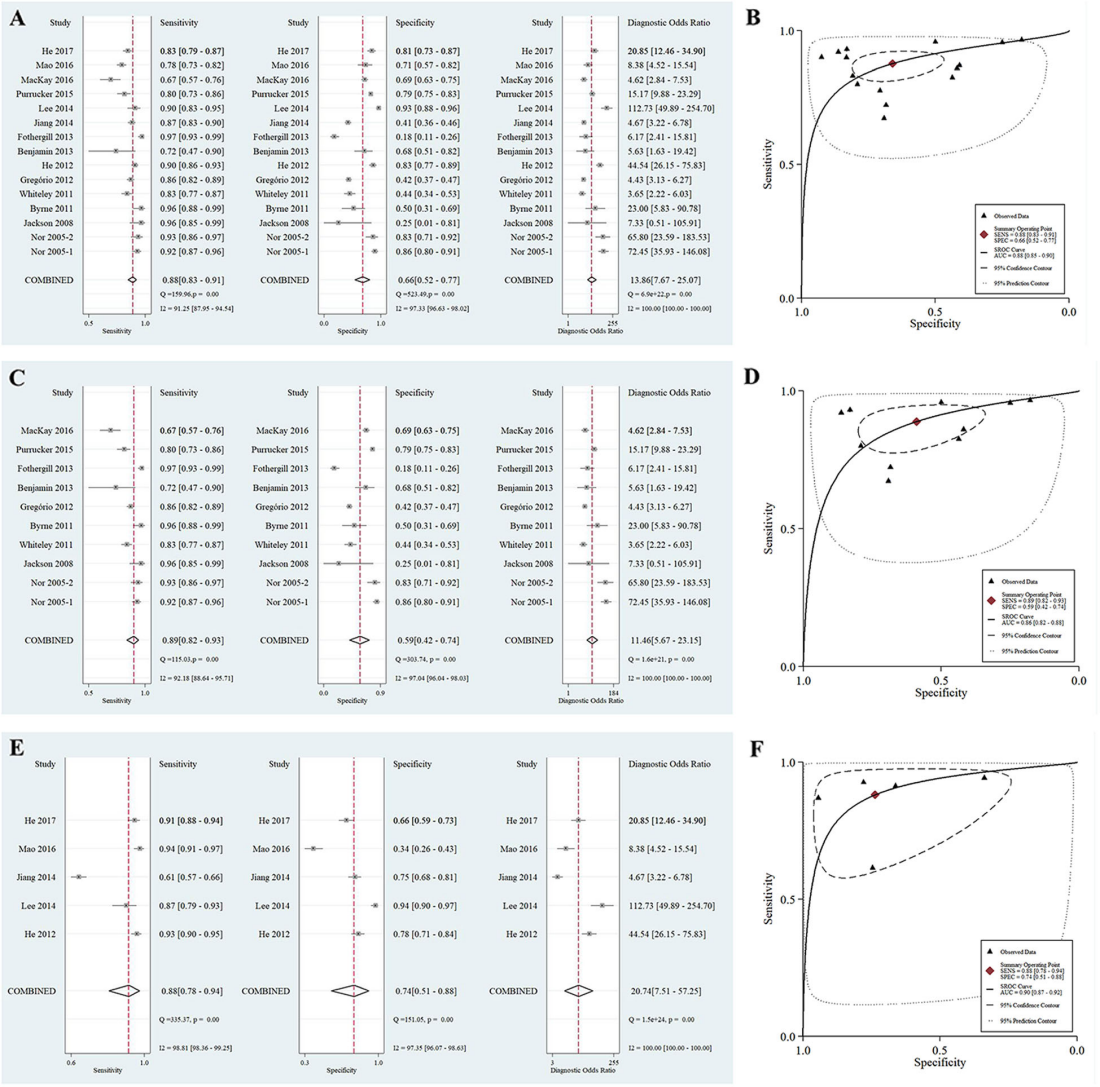
**The forest plot for evaluating the pooled sensitivity, specificity, diagnostic odds ratio, and the area under the curve for the performance of the ROSIER scale. a:** the forest plot for estimating the pooled sensitivity, specificity, and DOR in total population; **b:** the pooled AUC of the SROC in total population; **c:** the forest plot for estimating the pooled sensitivity, specificity, and DOR in Europe; **d:** the pooled AUC of the SROC in Europe; **e:** the forest plot for estimating the pooled sensitivity, specificity and DOR in Asia; **f:** the pooled AUC of the SROC in Asia **Abbreviations:** ROSIER = Recognition of Stroke in the Emergency Department; DOR = diagnostic odds ratio; AUC = area under the curve; SROC = summary receiver operating curve.

The pooled sensitivity, specificity, DOR and AUC were 0.88 (95% CI: 0.83–0.91), 0.66 (95% CI: 0.52–0.77), 13.86 (95% CI: 7.67–25.07) and 0.88 (95% CI: 0.85–0.90), respectively. (Fig. 2 a-b) Substantial heterogeneity existed in the pooled sensitivity (*I*^2^ = 91.25%, *p* < 0.001), pooled specificity (*I*^2^ = 97.33%, *p* < 0.001), and the pooled DOR (*I*^2^ = 100.00%, *p* < 0.001).

**Fig. 3:**
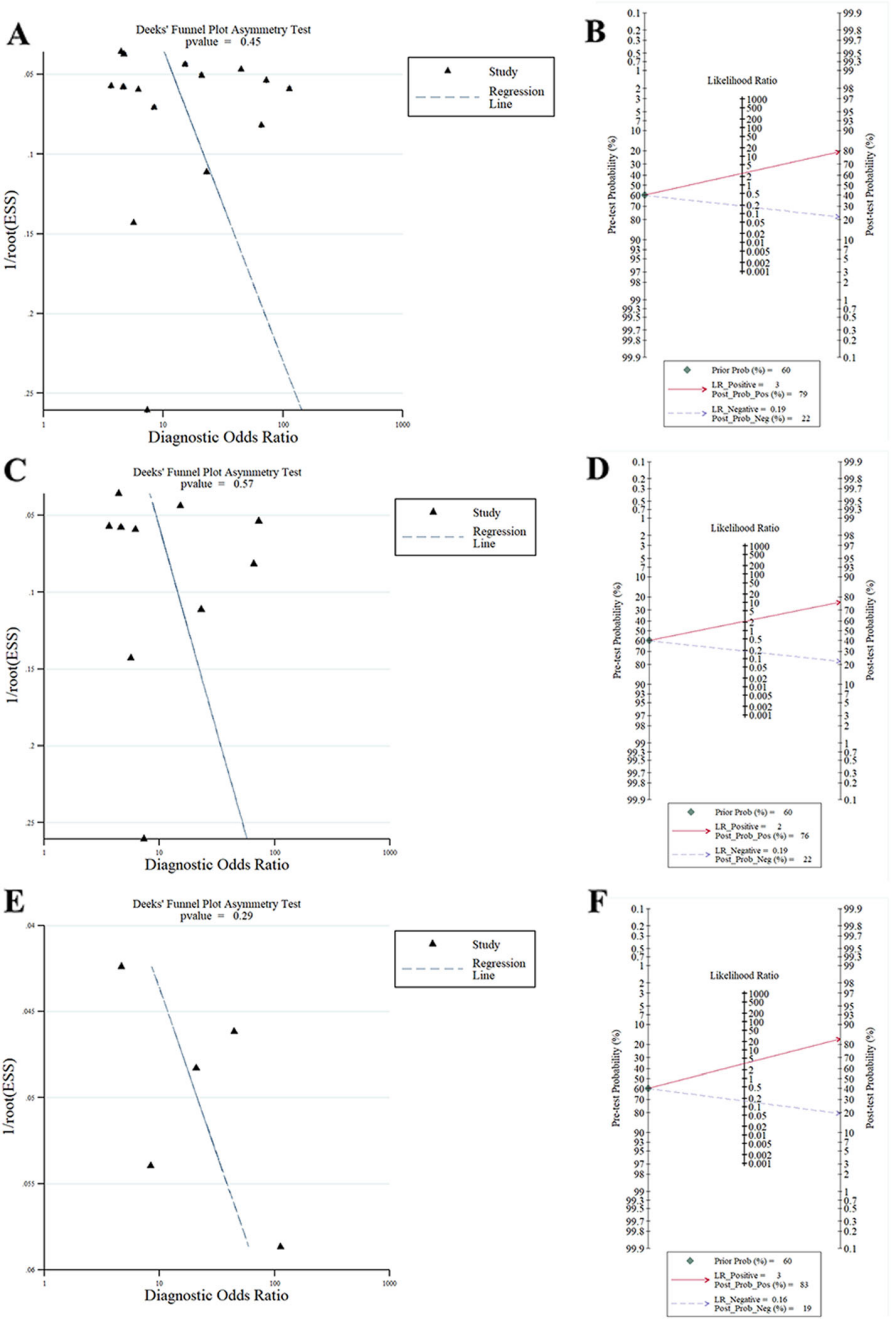
Deek’s **funnel plot and the Fagan nomogram for the performance of the meta-analysis. a:** The Deek’s funnel plot for estimating the publication bias in total population; **b:** the Fagan nomogram for ROSIER in total population; **c:** The Deek’s funnel plot for estimating the publication bias in Europe; **d:** the Fagan nomogram for ROSIER in Europe; **e:** The Deek’s funnel plot for determining the publication bias in Asia; **f:** the Fagan nomogram for ROSIER in Asia. **Abbreviations:** ROSIER = Recognition of Stroke in the Emergency Department

Sensitivity analysis showed that the pooled DOR was not significantly altered after omitting each study, which suggested the stability of the results. (Appendix file 1 A) Cumulative meta-analysis showed, with accumulating more data ranked by the publication year, the combined DOR was gradually decreased. (Appendix file 1 B) The pooled DOR was steadily improved, and the 95% CI became narrower by continually enlarging the sample size and the study quality. (Appendix file 1 C-D) The *p*-value for the slope of Deek’s funnel plot was 0.45, which indicated no publication bias. (Fig. 3 a) The Fagan nomogram showed, given the pre-test probability of 60.0%, the post-test likelihood was increased to 79% when the ROSIER was positive. In comparison, it was decreased to 22% when the ROSIER was negative. (Fig. 3 b).

Subgroup analysis showed there is a significant difference in the performance of the ROSIER scale between Europe and Asia population. The pooled sensitivity in Asia was lower than that in Europe. (Appendix file 2) When stratified by the geographic background, the pooled sensitivity, specificity, DOR and AUC in Europe were 0.89 (95% CI: 0.82–0.93), 0.59 (95% CI: 0.42–0.74), 11.46 (95% CI: 5.67–23.15) and 0.86 (95% CI: 0.82–0.88), respectively. (Fig. 2 c-d) Deek’s plot showed that no publication bias existed. (*P* for slope = 0.57, Fig. 3 c) Fagan nomogram showed, for the given pre-test probability of 60% for the suspected stroke patients, the post-test probability was 76 and 22% for the positive and negative results of the ROSIER scale, respectively. (Fig. 3 d) For the studies conducted in Asia, the pooled sensitivity, specificity, DOR and AUC were 0.88 (95% CI: 0.78–0.94), 0.74 (95% CI: 0.51–0.88), 20.74 (95% CI: 7.51–57.25) and 0.90 (95% CI: 0.87–0.92), respectively. The Deek’s funnel plot suggested no publication bias existed in Asia. (*p* for slope = 0.29, Fig. 3 e) Fagan nomogram showed, given a pre-test probability of 60%, the post-test probability increased to 83% when the ROSIER was positive, and it was reduced to 19% when negative. (Fig. 3 f).

Subgroup analysis also showed that the pooled sensitivity in emergency department [vs pre-hospital setting; 0.87 (95% CI: 0.83–0.91) vs 0.94 (95% CI: 0.89–0.99); *p* < 0.001], conducted by emergency physician [vs other medical personnel; 0.86 (95%CI: 0.81–0.91) vs 0.92 (95% CI: 0.86–0.97); *p* < 0.001] and higher than 1.0 male-to-female ratio [vs ≤ 1.0; 0.88 (95% CI: 0.83–0.93) vs 0.89 (95% CI: 0.84–0.93); *p* < 0.001] was significantly lower than their counterparts. However, there was no difference in the pooled specificity between them. Moreover, no differencewas detected in the diagnostic accuracy of ROSIER scale for different study design and study quality subgroups. (Appendix file 2).

## Discussion

The incidence of stroke is rising annually around the world. Early identification and treatment of stroke can improve treatment efficiency, mitigate sequelae, and even save lives [43]. Nor and coworkers conducted the ROSIER scale for helping the emergency physicians to identify the stroke patients efficiently, and this scale was also recommended by the National Institute for Health and Clinical Excellence [7, 44]. ROSIER was developed in the United Kingdom, and whether it was valid in other countries was seldom studied before. The present study showed that, after excluding each study conducted in other countries, the pooled DOR did not significantly change, which confirmed the external validation and the stability of the results. Furthermore, subgroup analysis showed that Asian populations had a relatively lower sensitivity and similar specificity compared with that in Europe. Thus, the ROSIER could also be widely used in Asia, especially in China, as most of the Asian studies included in this meta-analysis were conducted in China.

As shown in Appendix file 3, the ROSIER presented to include more items compared with the published stroke screening tools, such as Cincinnati Prehospital Stroke Scale (CPSS) [8], Face Arm Speech Test (FAST) [9], Los Angeles Prehospital Stroke Screen (LAPSS) [10] and the National Institute of Health stroke scale (NIHSS) [11]. Thus the ROSIER might have a relatively better performance in the stroke diagnosis, which was consistent with previous studies [13, 14, 25]. The ROSIER scale was firstly developed in the emergency department and was prospectively validated by emergency physicians [7]. The subgroup analysis showed that the performance of the ROSIER scale was comparable between prehospital settings and the emergency department. Moreover, results also suggested the other trained medical personnel present to have a significantly higher sensitivity and similar specificity compared with the emergency physicians in using the ROSIER scale. Thus, the ROSIER scale could be utilized in other workplaces and conducted by other trained investigators. It is an important finding, especially in China. Most of the stroke patients in China often occurred at home. Due to the limited health resources, not all of these patients could be transferred to the emergency department of a high-level hospital in time. According to the results in the present study, these patients could be firstly evaluated by the general practitioners in prehospital settings or community healthcare centers. The high-risk stroke patients should be transferred to the superior hospital as soon as possible. By establishing the community-hospital integrated model for the rapid treatment of stroke, and we can promote the diagnosis and treatment efficiency. Additionally, for the sake of the clinical applicability of the ROSIER in other work settings and investigators, it is of great importance to carry out comprehensive and systematic training to the medical personnel.

### Limitations

Although with the superiorities mentioned above, some issues also need to be focused. Under the condition that patients were in a coma state, and they were not companied with family members, the ROSIER score could not be accurately evaluated. If all of the items were scored “0”, that may result in a high false-negative rate. Although the sensitivity and specificity were relatively high, ROSIER could not wholly exclude the false-positive and false-negative rate. Thus, the ROSIER scale could just be regarded as a stroke screening tool, not the diagnostic criteria.

Moreover, substantial heterogeneities were detected to present across the studies. These heterogeneities were partly explained by factors such as geographic background, work setting, and investigators. However, it could not be markedly diminished and may affect the results to some extent. Although some studies tried to validate the performance of ROSIER, they were not included in the present study, due to the insufficient information for calculating the sensitivity and specificity with 95% CI of the ROSIER [36–42]. Thus, the results should be explained with caution.

## Conclusions

ROSIER is a valid and portable stroke screening scale. It can be used not only for the emergency physicians at the emergency department in Europe but also in extended prehospital workplaces with other fully trained medical personnel in Asia. Other high-quality validation studies with larger sample sizes and broader populations were needed to confirm the results and try to extend the application of the ROSIER scale in the future.

## Supplementary information

**Additional file 1.**

**Additional file 2.**

**Additional file 3.**

## Data Availability

All data generated or analyzed during this study are included in this published article.

## Abbreviations

AUC: Area under the curve
CI: Confidence interval
DOR: Diagnostic odds ratio
DOR: Diagnostic odds ratio
QUADASf: Quality Assessment of Diagnostic Accuracy Studies
ROSIER: Recognition of Stroke in the Emergency Department
SROC: Summary receiver operating characteristic curve
CPSS: Cincinnati prehospital stroke scale
FAST: Face arm speech test
LAPSS: Los Angeles prehospital stroke scale
NIHSS: National Institute of Health stroke scale.
