# Supplementary material for: A systematic review and meta-analysis to evaluate the diagnostic accuracy of recognition of stroke in the emergency department (ROSIER) scale
Source: BMC Neurol. 2020 Aug 18;20:304. doi: 10.1186/s12883-020-01841-x (PMC7433071; doi:10.1186/s12883-020-01841-x)
Supplement: Supplementary file 1 — Additional file 1. [file 12883_2020_1841_MOESM1_ESM.pdf]

A

Meta Analysis

Study removed	Publication year	Statistics with study removed: Point	Lower limit	Upper limit	Z-Value	p-Value
Nor 2005-1	2005	12.16	6.98	21.19	8.82	0.00
Nor 2005-2	2005	12.45	6.99	22.18	8.56	0.00
Jackson 2008	2008	14.09	7.87	25.22	8.90	0.00
Byrne 2011	2011	13.43	7.44	24.24	8.62	0.00
Whiteley 2011	2011	15.37	8.47	27.91	8.98	0.00
Gregório 2012	2012	15.19	8.26	27.92	8.76	0.00
He 2012	2012	12.56	7.15	22.08	8.79	0.00
Benjamin 2013	2013	14.60	8.07	26.44	8.85	0.00
Fothergill 2013	2013	14.62	8.03	26.62	8.78	0.00
Jiang 2014	2014	15.12	8.18	27.94	8.66	0.00
Lee 2014	2014	11.83	6.86	20.43	8.87	0.00
Purrucker 2015	2015	13.74	7.35	25.71	8.20	0.00
MacKay 2016	2016	15.10	8.22	27.74	8.74	0.00
Mao 2016	2016	14.38	7.79	26.54	8.53	0.00
He 2017	2017	13.39	7.29	24.58	8.37	0.00
		13.82	7.80	24.48	9.01	0.00

Diagnostic Odds Ratio with study removed

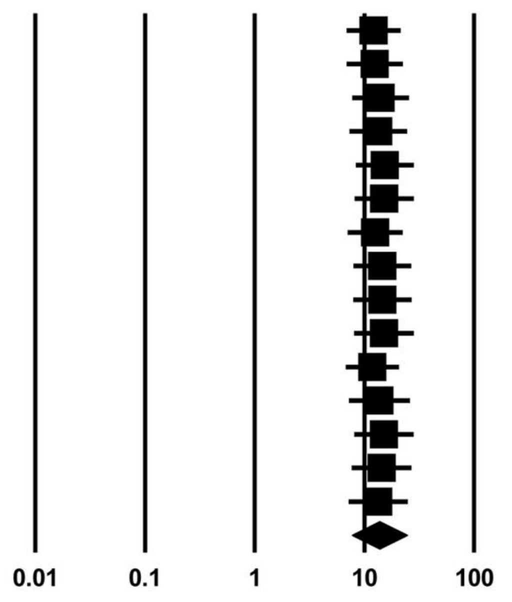

Meta Analysis

B

Meta Analysis

Study	Publication year	Cumulative statistics: Point	Lower limit	Upper limit	Z-Value	p-Value
Nor 2005-1	2005	72.45	35.93	146.08	11.97	0.00
Nor 2005-2	2005	70.26	39.38	125.35	14.40	0.00
Jackson 2008	2008	59.63	28.80	123.46	11.01	0.00
Byrne 2011	2011	48.28	23.70	98.37	10.68	0.00
Whiteley 2011	2011	20.79	4.17	103.65	3.70	0.00
Gregório 2012	2012	15.53	4.79	50.38	4.57	0.00
He 2012	2012	18.39	6.06	55.85	5.14	0.00
Benjamin 2013	2013	15.95	5.77	44.07	5.34	0.00
Fothergill 2013	2013	14.31	5.70	35.93	5.66	0.00
Jiang 2014	2014	12.57	5.77	27.39	6.37	0.00
Lee 2014	2014	15.52	6.91	34.87	6.64	0.00
Purrucker 2015	2015	15.48	7.59	31.56	7.54	0.00
MacKay 2016	2016	13.94	7.24	26.87	7.87	0.00
Mao 2016	2016	13.39	7.29	24.58	8.37	0.00
He 2017	2017	13.82	7.80	24.48	9.01	0.00
		13.82	7.80	24.48	9.01	0.00

Cumulative Diagnostic Odds Ratio

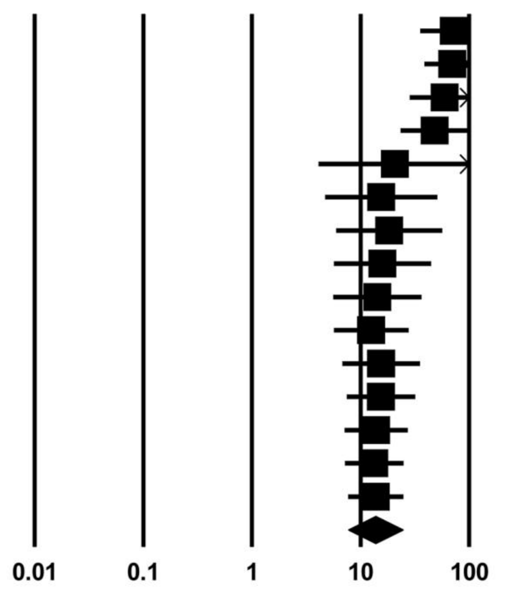

Meta Analysis

C

Meta Analysis

Study	Sample size	Cumulative statistics: Point	Lower limit	Upper limit	Z-Value	p-Value
Jackson 2008	50	7.33	0.51	105.91	1.46	0.14
Benjamin 2013	56	5.90	1.92	18.14	3.10	0.00
Byrne 2011	100	10.24	3.95	26.57	4.78	0.00
Nor 2005-2	160	18.12	5.02	65.35	4.43	0.00
Fothergill 2013	295	14.00	4.67	41.98	4.71	0.00
Lee 2014	312	20.65	6.23	68.40	4.95	0.00
Nor 2005-1	343	25.78	9.49	70.05	6.37	0.00
Whiteley 2011	356	18.98	5.93	60.71	4.96	0.00
MacKay 2016	380	15.97	5.82	43.82	5.38	0.00
Mao 2016	416	14.89	6.22	35.64	6.06	0.00
He 2017	468	15.42	7.16	33.20	6.99	0.00
He 2012	540	17.01	8.19	35.34	7.60	0.00
Purrucker 2015	640	16.87	8.92	31.88	8.70	0.00
Jiang 2014	715	15.19	8.26	27.92	8.76	0.00
Gregório 2012	779	13.82	7.80	24.48	9.01	0.00
		13.82	7.80	24.48	9.01	0.00

Cumulative Diagnostic Odds Ratio

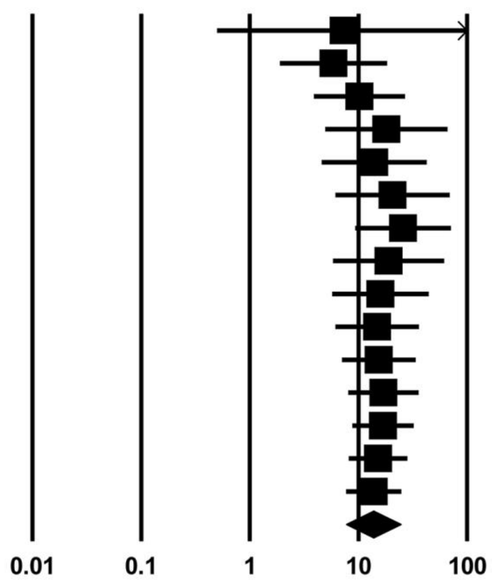

Meta Analysis

D

Meta Analysis

Study	Study quality	Cumulative statistics: Point	Lower limit	Upper limit	Z-Value	p-Value
MacKay 2016	3	4.62	2.84	7.53	6.16	0.00
Jackson 2008	4	4.69	2.91	7.58	6.32	0.00
Benjamin 2013	4	4.81	3.07	7.52	6.88	0.00
Fothergill 2013	5	5.03	3.36	7.54	7.84	0.00
Nor 2005-1	6	10.12	2.69	38.00	3.43	0.00
He 2012	6	13.43	4.19	42.97	4.38	0.00
Jiang 2014	6	11.36	4.23	30.52	4.82	0.00
Purrucker 2015	7	11.92	5.32	26.70	6.02	0.00
Mao 2016	7	11.46	5.63	23.32	6.72	0.00
Nor 2005-2	8	13.52	6.72	27.17	7.31	0.00
Byrne 2011	8	14.07	7.24	27.34	7.80	0.00
Whiteley 2011	8	12.38	6.58	23.31	7.80	0.00
He 2017	8	12.95	7.21	23.26	8.57	0.00
		12.95	7.21	23.26	8.57	0.00

Cumulative Diagnostic Odds Ratio

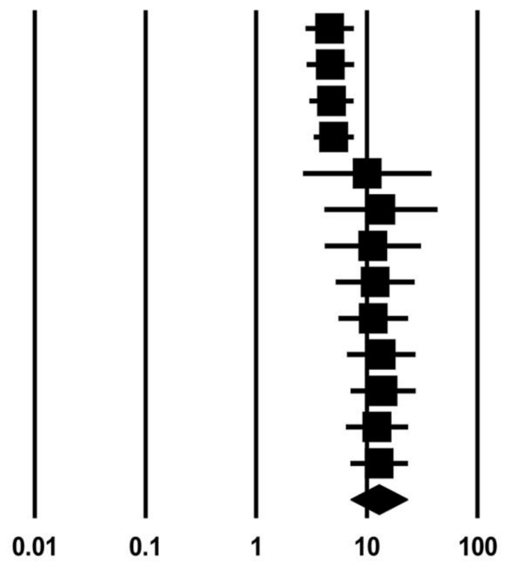

Meta Analysis

Appendix file 1: Sensitivity and cumulative meta-analysis for estimating the performance of ROSIER scale.

A: Sensitivity analysis for the performance of the ROSIER scale; **B:** Cumulative meta-analysis for estimating the performance when ranking by the publication year; **C:** Cumulative meta-analysis for estimating the performance when ranking by the sample size. **D:** Cumulative meta-analysis for estimating the performance when ranking by the study quality. **Abbreviations:** ROSIER= Recognition of Stroke in the Emergency Department.
