# Supplementary material for: A systematic review and meta-analysis to evaluate the diagnostic accuracy of recognition of stroke in the emergency department (ROSIER) scale
Source: BMC Neurol. 2020 Aug 18;20:304. doi: 10.1186/s12883-020-01841-x (PMC7433071; doi:10.1186/s12883-020-01841-x)
Supplement: Supplementary file 2 — Additional file 2. [file 12883_2020_1841_MOESM2_ESM.pdf]

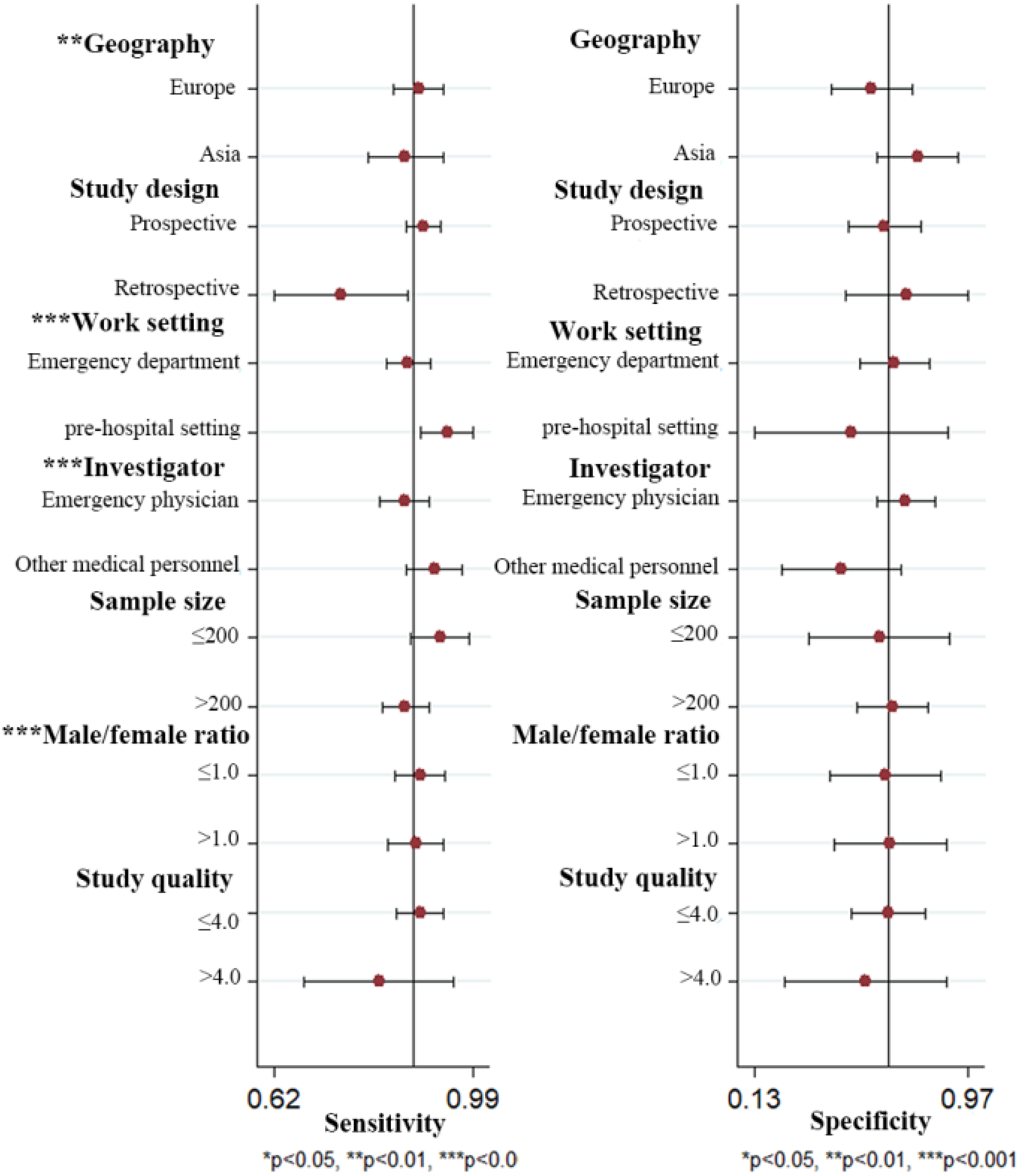

Appendix file 2: The forest plot for subgroup analysis by analyzing the difference in the pooled sensitivity and specificity of Recognition of Stroke in the Emergency Department scale.
