# Supplementary material for: A systematic review and meta-analysis to evaluate the diagnostic accuracy of recognition of stroke in the emergency department (ROSIER) scale
Source: BMC Neurol. 2020 Aug 18;20:304. doi: 10.1186/s12883-020-01841-x (PMC7433071; doi:10.1186/s12883-020-01841-x)
Supplement: Supplementary file 3 — Additional file 3. [file 12883_2020_1841_MOESM3_ESM.pdf]

Appendix file 3: Comparison of the difference in the items for published stroke screening scales.

Items	ROSIER	CPSS	FAST	LAPSS	NIHSS
**Disease history**	√	×	×	√	×
**Facial muscle movement**	√	√	√	√	√
**Bodily-kinestic**	√	√	√	√	√
**Language dysfunction**	√	√	√	×	√
**Visual field defect**	√	×	×	×	√
**Pathoglycemia**	√	×	×	√	×
**Consciousness, sensation, feel, ataxia**	×	×	×	×	√

Abbreviations: ROSIER= Recognition of Stroke in the Emergency Department; CPSS= Cincinnati prehospital stroke scale; FAST= face arm speech test; LAPSS= Los Angeles prehospital stroke scale; NIHSS= National Institute of Health stroke scale.
